# Comparison of sequence-dependent tiling array normalization approaches

**DOI:** 10.1186/1471-2105-10-204

**Published:** 2009-06-30

**Authors:** Ho-Ryun Chung, Martin Vingron

**Affiliations:** 1 Max-Planck-Institut für molekulare Genetik, Department of Computational Molecular Biology, Ihnestraße 73, 14195 Berlin, Germany

## Abstract

**Background:**

The detection of enriched DNA or RNA fragments by tiling microarrays has become more and more popular. These microarrays contain a high number of small probes covering genomic loci. However, to achieve high coverage the probe sequences cannot be selected for their hybridization properties. The affinity of the probes towards their targets varies in a sequence-dependent manner. In order to remove this bias a number of approaches have been developed and shown to increase the detection of enriched DNA or RNA fragments. However, these approaches also employ a peak detection algorithm that is different from the one used previously. Thus, it seems possible that the enhancement of detection is due to the peak detection algorithm rather than the sequence-dependent normalization.

**Results:**

We compared three different sequence-dependent probe level normalization procedures to a naïve sequence-independent normalization technique. In order to achieve maximal comparability, we used the normalized intensity values as input to a single peak detection algorithm. A so-called "spike-in" data set served as benchmark for the performance. We will show that the sequence-dependent normalization procedures do not perform better than the naïve approach, suggesting that the benefit of using these normalization approaches is limited. Furthermore, we will show that the naïve approach does well, because it effectively removes the sequence-dependent component of the measured intensities with the help of the control hybridization experiment.

**Conclusion:**

Sequence-dependent normalization of microarray data hardly improves the detection of enriched DNA or RNA fragments. The "success" of the sequence-independent naïve approach is only possible due to the control experiment and requires proper scaling of the measured intensities.

## Background

Tiling microarrays are widely used to detect enriched DNA or RNA fragments generated by, for example, chromatin immunoprecipitation or expression profiling. Tiling microarrays allow for detecting simultaneously the enrichment of many fragments on a genome wide scale in an unbiased manner. This is facilitated by a high number of small probes, usually in the range of 25 to 60 nucleotides long. In order to achieve high coverage of the genomic sequence it is not possible to select the probes for their hybridization properties. Thus, the affinity of the probes towards their targets varies in a sequence-dependent manner. Recently, a number of approaches have been developed in order to remove the bias introduced by the sequence-dependent hybridization properties of the probes (e.g. methods dealing with tiling microarrays MAT [1]; MA2C [2]; PMT [3]; [4] and methods dealing with gene expression arrays [5,6]). These approaches have been shown to enhance the ability to detect enriched DNA fragments compared to other methods. However, MAT, MA2C and PMT not only normalize the measured intensities by removing the sequence-dependent bias but also implement a peak calling procedure that is different to other methods. Hence, the improved ability to detect enriched regions may be due to the peak calling procedure.

In order to test this possibility, we employed recently published data obtained by so-called spike-in experiments [7]. We compared the effect of sequence-dependent probe-level normalization on the ability to detect enriched genomic regions, whose genomic locations we know beforehand. Specifically, we tested three normalization methods: MAT [1], MA2C [2] and an updated version of PMT developed by us [3]. As control, we normalized the measured intensities by subtracting the mean and scaling the resulting deviations from the mean such that they have a variance of 1 on a logarithmic scale, referred to as standard normal normalization (SNN). To separate the effects of the normalization procedure from peak calling we used the normalized intensities as input for a single peak-calling algorithm. To our surprise we found that the three sequence-based normalization methods showed an almost identical performance compared to SNN. Thus, the sequence-dependent probe level normalization has only a minor impact on the ability to detect enriched genomic regions. We propose that the intensities measured for the control samples are the best guide to remove sequence-dependent biases.

## Results and Discussion

### Comparison of normalization methods

In order to assess the effect of sequence-dependent probe-level normalization on the ability to detect enriched genomic regions, we analyzed the data generated by so-called spike-in experiments [7]. The spike-in samples contained about 100 DNA fragments of approximately 500 base pair length at different concentrations. Otherwise the spike-in samples were exactly the same as the control samples consisting of sonicated whole genome DNA. Spike-in and control samples were either directly labeled and hybridized to tiling microarrays or were diluted such that these samples required amplification prior to hybridization. Moreover, different microarray platforms were used, namely tiling microarrays manufactured by Affymetrix, Agilent and NimbleGen covering the ENCODE regions.

We used three sequence-dependent (MAT [1]; MA2C [2]; PMT [3]; this study) and one sequence-independent normalization procedure (standard normal normalization (SNN); this study). We implemented all four methods in R [8] in order to assure that the same input data is used for normalization (see Methods). We selected for each of the tiling microarray platforms datasets, which were performed on both amplified and unamplified samples, namely the ones provided by the Struhl and Gingeras (Affymetrix), Farnham and Green (NimbleGen) and McCuine (Agilent) labs. We did so because we wanted to compare the effect of the amplification step on the ability to detect enriched genomic regions.

We used the raw data as input for the four normalization strategies. The resulting normalized intensities (on a logarithmic scale) were subsequently used in a single peak calling procedure adapted from Johnson *et al*. (2006) [1]. Thereafter, the predicted spike-in regions were ordered by their enrichment score and represented by the start and end coordinates of the scored window. We considered predictions, which overlapped the spike-in region as true positive and all others as false positive. In order to assure that each spike-in region is counted only once as true positive prediction, we removed all lower ranking predictions falling within the boundaries of already predicted spike-in regions.

We adopted the same assessment approach as in the original study ([7], see also Methods). Briefly, we determined the cumulative number of true and false positives by traversing the rank ordered list of the predictions. These numbers were divided by the total number of spike-in regions (98 for the unamplified and 100 for the amplified samples). We determined the area under this ROC (receiver operating characteristic)-like curve (AUC). Here, the AUC is bounded by zero (almost random prediction) and one (perfect performance, i.e. 100% sensitivity and 100% specificity). In the original study, the AUC is determined only in the interval between 0 and 10% false positives and than multiplied by 10 (Li personal communication), i.e. there are only about 10 false positive predictions allowed. In order to compare our results to the findings reported by the original study, we adhered to this procedure.

**Figure 1:**
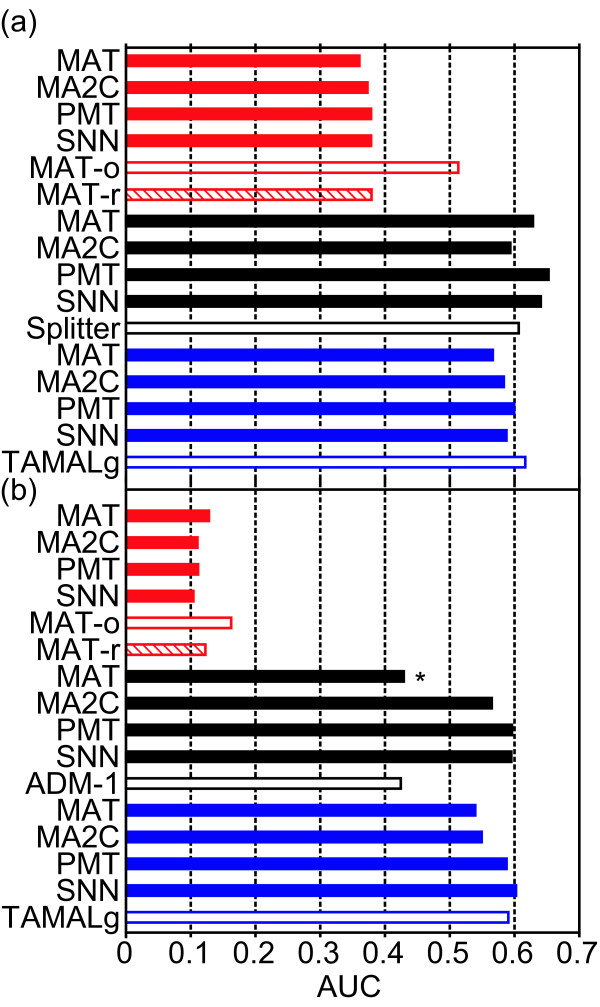
**Performance of normalization procedures**. For each platform and normalization method the modified AUC values are shown. The open bar indicates the best performing algorithm as found in the original study [7]. The red hatched bar indicates the results from our rerun experiment. MAT-o refers to the result of the MAT method in the original study and MAT-r refers to the results of our rerun experiment. In red the results for the Affymetrix platform (Struhl and Gingeras labs), in black the results for the Agilent platform (Farnham and Green labs) and in blue the results for the NimbleGen platform (McCuine lab). (a) Results using the unamplified samples. (b) results using the amplified samples. The asterisk marks the much lower AUC value using MAT on the intensities measured for the amplified sample of the McCuine lab (Agilent platform).

The results of this analysis are summarized in Figure 1. We included as a reference the AUC of the best performing algorithm using the data of each lab. Note that we used the ranked list for each of these algorithms and calculated the AUC values by our method, i.e. we removed multiple hits to the same spike-in region. Irrespective of the platform and the sample type we found that the three sequence-dependent normalization methods perform very similarly to each other. Moreover, we observed that none of them performed better than the sequence-independent SNN approach. The AUC values of each of the four methods compared to the best performing one were in almost all cases only 0.06 smaller. However, our implementation of the MAT method performs worse than the original implementation using both the unamplified (Figure 1a, open red bar) and amplified sample (Figure 1b, open red bar). We recomputed ranked lists using the original implementation and the same input data provided to our implementation. Performance analysis revealed that AUC values obtained by the recomputed ranked lists are very similar to our implementation of MAT (Figure 1 hatched red bar), suggesting that different input data and/or parameters led to the different results rather than errors in our implementation of the MAT approach. The other exception was the AUC value obtained by MAT in the amplified sample hybridized to an Agilent microarray by the McCuine lab (Figure 1b marked by an asterisk). Given that the confidence interval of the AUC value has been determined to be around ± 0.07 [7], we conclude that sequence-dependent normalization does not increase the ability to detect enriched genomic regions.

In contrast to our conclusion, earlier studies reported that sequence-dependent probe-level normalization increases the ability to identify enriched genomic regions [1-3]. We think that the major performance increase is not due to the normalization procedure but rather due to the peak calling approach utilized after pre-processing the raw data. Peaks are predicted by calculating the trimmed mean of signal values in a sliding window, where the 10% highest and lowest values are discarded. The resulting trimmed mean is multiplied by the square root of probes contained in the corresponding window (see Methods and Johnson *et al*. (2006)[1]). We refer to this approach as trimmed mean, which seems to perform very well compared to other methods [1].

In order to check whether the trimmed mean approach performs exceptionally well, we compared the performance of this approach to algorithms that have been reported to perform best for the respective dataset [7]. In almost all cases the trimmed mean procedure received AUC values within the aforementioned confidence interval of the best performing algorithm. Only for the amplified sample hybridized to the Agilent microarray by the McCuine lab the results for MA2C, PMT and SNN were much better than for the reported ADM-1 algorithm. However, we note that most of the ranked lists reported by the original study contained only a single chromosomal coordinate per predicted spike-in region, such that the AUC results may not be directly comparable. Irrespective of this concern, we conclude that the trimmed mean procedure is not better but also not worse than other existing methods.

**Figure 2:**
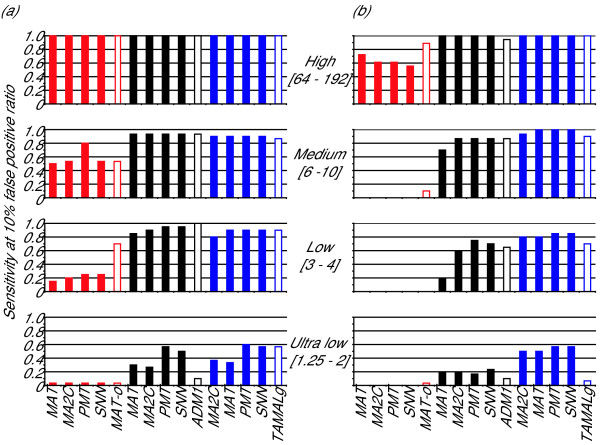
**PCR amplification does not affect the detection**. Shown are the recall rates (maximal false positive rate of 10%) of the spike-in fragments in four different concentration categories: high(enrichment between 64 and 192 fold), medium (enrichment between 6 and 10 fold), low (enrichment between 3 to 4 fold) and ultra low (enrichment between 1.25 and 2). In red the results for the Affymetrix platform (Struhl and Gingeras labs), in black the results for the Agilent platform (Farnham and Green labs) and in blue the results for the NimbleGen platform (McCuine lab). (a) using the unamplified samples. (b) using the amplified samples.

Finally, we compared the performance of the four normalization methods in the two types of samples, i.e. amplified or unamplified. We reasoned that we could minimize the effects of different experimental procedures by comparing datasets generated by the same lab. Amplification had a negative impact on the ability to detect enriched regions only in the experiment employing Affymetrix tiling arrays, while it had no effect using Agilent or NimbleGen arrays (compare Figure 1a and 1b). The decrease in specificity was due to a decline of the recall rate across all spike-in concentrations in the experiments involving Affymetrix tilling arrays. Comparison of the concentration-dependent recall rates for the amplified and unamplified samples interrogated by Agilent and NimbleGen tiling arrays showed no such behavior (Figure 2). However, we think that this negative effect of amplification cannot be attributed to the Affymetrix platform, as datasets generated by the Brown lab interrogating an amplified sample by another Affymetrix tiling array yielded comparable results to the ones obtained by Struhl and Gingeras using an unamplified sample (data not shown; [7]). The difference between the two Affymetrix tiling arrays is the number of probes spotted on the array, i.e. the spatial resolution. The array used by the Struhl and Gingeras lab had ~3 times more probes than the one used by the Brown lab. We speculate that PCR amplification results in a preferential enrichment of short DNA fragments, which are more easily mistaken for real enrichment in tiling arrays with higher spatial resolution. Thus, we conclude that amplification *per se *has no negative impact on the ability to detect enriched genomic regions, but limits the detection of these loci using tiling arrays with high spatial resolution. Furthermore, we speculate that the lower performance of the Affymetrix array interrogating the unamplified sample has a similar explanation. Based on this assumption we propose that tiling arrays with high spatial resolution require a refined peak calling procedure that accounts for the possibility of false positive detection of small DNA fragments.

### Standard normal normalization

Contrary to our expectation that sequence-dependent probe-level normalization increases the ability to detect enriched genomic regions, we found no evidence for such an effect. Our "control" normalization procedure, SNN, performed in most of the cases similar to the three sequence-dependent normalization approaches, suggesting that it effectively removes biases due the sequence composition. The SNN procedure consists of three steps: (1) transformation of the intensities to a logarithmic scale (log intensity); (2) subtraction of the mean log intensity, which is intended to remove systematic shifts in the intensity distribution between arrays (and/or channels for two color arrays); (3) division by the standard deviation, which is intended to remove differences in the intensity scales.

**Figure 3:**
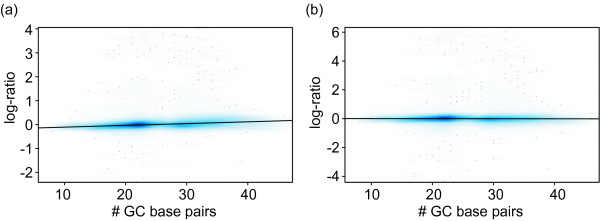
**SNN removes sequence-dependent biases using the control experiment**. Scatter plot with the GC content of the probes on the x-axis and the log-ratio between spike-in and control hybridization; shades of blue indicate the density of points, the darker the more points. The line indicates a linear fit between the GC content and the log-ratio. (a) The ratio between the spike-in and control intensities on a logarithmic scale centered on the respective mean log intensities. The GC content and the log-ratio correlate with a Pearson correlation coefficient r = 0.26. (b) The same ratio after division by the standard deviation. Here, the GC content and the log-ratio are not correlated anymore (r = -0.02).

These operations are in general not able to remove the sequence-dependent biases of the intensities measured by each individual array and/or channel. The correlation between the SNN normalized intensities and, for example, the GC content remained unchanged. The picture dramatically changes if we consider the dependency between the ratio of the intensities measured for the spike-in and the control samples on a logarithmic scale (log-ratio) and the GC content. Here, we found that the division of the standard deviation effectively removed most of the sequence inherent biases observed by only subtracting the mean log-intensity (compare Figure 3a and 3b). Thus, the control hybridization is the best guide to remove sequence-dependent biases, but it has to be properly rescaled in order to display its normalizing character.

## Conclusion

We have demonstrated that sequence-dependent normalization hardly improves the detection of enriched genomic regions. We were able to draw this conclusion by utilizing spike-in experiments, which emphasize the requirement of clear-cut benchmarks to recover the advantages and disadvantages of analysis approaches. We found that the "success" of the sequence-independent normalization method, referred to as SNN, can be attributed to the normalizing character of the control experiment, which depends on a proper scaling of the measured intensities.

## Methods

### Raw data for spike-in Experiments

We downloaded the raw data from NCBI GEO [9]. For the amplified samples processed and hybridized by the Struhl and Gingeras lab we downloaded 6 raw CEL files corresponding to the identifiers [GEO:GSM249008 to GSM249010] (3 spike-in samples), [GEO:GSM249011 to GSM249013] (3 control samples); by the Farnham and Green lab 6 raw files corresponding to the identifiers [GEO:GSM254805 to GSM254807] (each channel separately); by the McCuine lab 2 raw files corresponding to the identifiers [GEO:GSM248658 and GSM248666] (both channels). For the unamplified samples processed and hybridized by the Struhl and Gingeras lab we downloaded 12 raw CEL files corresponding to the identifiers [GEO:GSM248996 to GSM248998] and [GEO:GSM249002 to GSM249004] (6 control samples), [GEO:GSM248999 to GSM249001] and [GEO:GSM249005 to GSM249007] (6 spike-in samples); by the Farnham and Green lab 8 raw files corresponding to the identifiers [GEO:GSM254930, GSM254971 to GSM254973] (each channel separately); by the McCuine lab 2 raw files corresponding to the identifiers [GEO:GSM248654 and GSM252509] (both channels).

The array designs and probe sequences were taken from files corresponding to the identifiers [GEO:GPL6129] (Affymetrix, Struhl and Gingeras lab), [GEO:GPL4559] (NimbleGen, Farnham and Green lab) and [GEO:GPL6189] (Agilent, McCuine lab).

### Sequence-dependent probe-level normalization

MAT was originally implemented by Johnson et al. (2006) [1]. As reference we downloaded the source code of MAT and re-implemented the algorithm in R [8] with only one modification, i.e. we removed the term which models the effect of the number of times a probe occurs in the genome (variable named c_i _in [1]). We estimated the parameters of the model using a standard linear model fitting procedure. Using this model we computed the expected intensity due to non-specific hybridization and the residuals of the measured intensities. The probes were then divided into 3000 bins based on the expected intensity. The residual intensity of each probe was then divided by the standard deviation of the bin the probe belongs to [1]. We reran the original MAT implementation (version 2.09232006) on the input data using the following parameters: Bandwidth = 200, MaxGap = 200, MinProbe = 10, Var = 0, Pvalue = 1e-5.

MA2C was originally implemented by Song et al. (2007) [2]. As reference we downloaded the source code of MA2C (REF) and re-implemented the algorithm in R [8]. Specifically, we implemented the so-called robust version of their algorithm. Here, the probes are grouped into bins with equal GC content and for each GC content bin the mean intensity and the standard deviation per channel is computed and is used to estimate the enrichment of sequences corresponding to a probe [2]. We modified the algorithm such that it can also be used to normalize data from single color arrays. Furthermore, we compared each spike-in sample to each control sample.

The basic approach of PMT is outlined in Chung *et al*. (2007) [3]. We did not change the estimation procedure of the free energy change due to unspecific hybridization. However, we substituted the linear model for the estimation of the intensity due to unspecific hybridization with a non-linear model, which is referred to as Hill equation:



Here, the *I*_*i *_corresponds to the intensity measured for probe *i *and *ΔG*_*i *_corresponds to our estimated free energy change due to unspecific hybridization. *I*_0_, *β *and *α *are parameters that have to be estimated. We can however attribute some meaning to two of these parameters, namely *I*_0 _corresponds to the average intensity of the sample and *α *corresponds to the logarithm of average concentration (or better activity) of free probes.

We would like to note that this model could be approximated by a linear model if exp [-*β ΔG*_*i *_- *α*)] is very large compared to 1. This is the case when *α *is very small, i.e. if the concentration of free probe is very small. If true this suggests that the higher the concentration of sample gets the higher will be the effect of unspecific hybridization. Thus, we predict that if one uses too much sample one measures mainly the physical properties of the probes, while by using fewer samples one can minimize the effect of unspecific hybridization.

### Standard normal normalization

The standard normal normalization (SNN)is very similar to quantile normalization [10]. However, SNN normalizes each array and/or channel separately and the normalized intensities on a logarithmic scale are taken directly without further processing. It involves three steps: (1) the raw intensities are transformed to a logarithmic scale, the resulting values are referred to as log intensities; (2) the mean log-intensity is subtracted from the log intensities and (3) divided by the standard deviation.

### Peak detection

We adapted the peak calling procedure proposed by Johnson *et al*. (2006) [1]. Specifically, we computed the mean of the normalized intensities of probes mapping to a sliding window of 500 base pairs. We discarded the 10% lowest and 10% highest intensities, if the number of probes within a window was more than nine and requested that each window contained at least three probes. The window means were either separately calculated for each array and/or channel (for the MAT, PMT and SNN normalized intensities), or for the combined values (for the MA2C normalized intensities). We summed the window means corresponding to the spike-in samples and subtracted the sum of the window means of the control samples. The resulting score was finally multiplied by the square root of the number of probes within the window. In a final step we extracted local score maxima, such that none of the overlapping windows had a score higher than the chosen one.

### Performance assessment

We adapted the assessment scheme of Johnson *et al*. 2008 [7]. Here, the predicted windows were ranked according to their score and it was checked whether the window overlapped any of the 98 (unamplified) or 100 (amplified) fragments. If it overlapped, we scored it as true positive, if not, as false positive. In order to assure that each fragment is only predicted once, we discarded all further overlapping predictions, i.e. they were neither counted as true nor false positive. We calculated the true positive rate as the number of true positives divided by the number of spike-in fragments and the false positive rate as the number of false positives divided by the number of spike-in fragments. As a performance measure we determined the area under the ROC-like curve (AUC), where we plotted the true positive rate against the false positive rate as defined above. We computed the AUC up to a false positive rate of 10%, i.e. 10 false positives and multiplied it by 10 to arrive at AUC values between 0 (random prediction) and 1 (perfect prediction).

In order to check whether the sensitivities were dependent on the enrichment of the spike-in fragments, we computed the percentage of correctly predicted spike-ins in four different enrichment classes with maximally 10% false positive predictions, namely high(enrichment between 64 and 192 fold), medium (enrichment between 6 and 10 fold), low (enrichment between 3 to 4 fold) and ultra low (enrichment between 1.25 and 2).

## Authors' contributions

HRC wrote the analysis software, conducted the analysis and drafted the manuscript. MV drafted the manuscript. All authors read and approved the final manuscript.
